# Icatibant for Angiotensin-Converting Enzyme Inhibitor-Induced Angioedema in Intubated Patients: Case Series and Literature Review

**DOI:** 10.1155/2018/8081607

**Published:** 2018-03-04

**Authors:** Erin K. Yeung, Haritha Saikumar, Jose Castaneda-Nerio, Sandra G. Adams, Mark Wong

**Affiliations:** ^1^Department of Pharmacy Services, South Texas Veterans Health Care System, 7400 Merton Minter, San Antonio, TX 78229, USA; ^2^Pharmacotherapy Education & Research Center, The University of Texas Health San Antonio, 7703 Floyd Curl Dr., MSC 6220, San Antonio, TX 78229-3900, USA; ^3^Medicine Service, Pulmonary/Critical Care Division, South Texas Veterans Health Care System, 7400 Merton Minter (111E), San Antonio, TX 78229, USA; ^4^Department of Medicine, Pulmonary/Critical Care Division, The University of Texas Health San Antonio, 7703 Floyd Curl Dr., MSC 7885, San Antonio, TX 78229-3900, USA

## Abstract

**Purpose:**

A case series of icatibant use in intubated patients with angiotensin-converting enzyme inhibitor- (ACEI-) induced angioedema is presented along with a relevant literature review and recommendations for utilization.

**Summary:**

Three intubated patients admitted to the intensive care unit for ACEI-induced angioedema were treated with icatibant. A literature search identified one controlled study and four case reports describing the use of icatibant in intubated ACEI-induced angioedema patients.

**Conclusion:**

Icatibant administration in intubated patients may be beneficial in decreasing time to extubation and length of intensive care unit stay. In the three cases described, icatibant administration did not appear to elicit a response in intubated patients, which has been described in previous case reports. For clinicians considering icatibant in the treatment of ACEI-induced angioedema, earlier administration upon arrival to the ED or immediately upon arriving to the intensive care unit is strongly advised. The suggested benefit of icatibant in intubated ACEI-induced angioedema patients should be verified by randomized clinical trials and cost-benefit analyses should be performed at individual institutions.

## 1. Introduction

ACEI-induced angioedema accounts for approximately one-third of angioedema cases treated in emergency departments (ED) [1]. It is estimated to occur in approximately 0.68% of patients who take an FDA-approved ACEI [2]. Factors contributing to higher risk for ACEI-induced angioedema include: African ancestry; age > 65 years; female gender; history of smoking; heart failure; treatment with statins, aspirin, or sirolimus; and history of drug rashes, seasonal allergies, or previous angioedema [1].

Icatibant is a bradykinin 2 (B2) receptor antagonist that was FDA-approved in 2011 for the treatment of hereditary angioedema [3]. Due to ACEI-induced bradykinin degradation inhibition and subsequently increased B1/B2 activation, icatibant has also been used as an off-label medication for the treatment of ACE-induced angioedema [2]. Evidence on off-label icatibant usage for ACEI-induced angioedema includes case reports, observational studies, and three randomized, controlled trials [2, 4, 5]. In 2015, Baş et al. treated 13 Caucasian adults presenting to the ED for ACEI-induced angioedema of the upper aerodigestive tract with icatibant and found a significantly shorter time to complete resolution of ACEI-induced angioedema compared to combination glucocorticoid plus antihistamine (prednisolone plus clemastine) therapy within 10 hours of symptom onset. The median time to onset of symptom relief was also significantly shorter with icatibant than with standard therapy (2 hours versus 11.7 hours). None of the patients required intubation/tracheostomy. Only one patient in the placebo group experienced no improvement after six hours of initial therapy and required rescue icatibant with prednisolone intervention [4]. Therefore, the authors concluded that icatibant can be used in patients with ACEI-induced angioedema within 10 hours of symptom onset to prevent respiratory intervention (intubation or tracheostomy). On the contrary, Straka et al. treated 13 patients (including Caucasian and African American) with ACEI-induced angioedema within six hours of presentation with icatibant and did not find any difference in the time to symptom resolution compared to placebo. One patient treated with icatibant could not complete the visual analog scale used to measure resolution of symptoms due to intubation and was subsequently excluded from the final analysis [5]. Similarly, a randomized, controlled, double-blind trial by Sinert et al. did not find a difference in time to meeting discharge criteria or time to onset of symptom relief between icatibant and placebo treatment for ACEI-induced angioedema in 121 patients [6].

While evidence exists to suggest significantly faster resolution of ACEI-induced angioedema with icatibant treatment, nearly all patients analyzed in randomized, controlled trials were not intubated [4–6]. Therefore, the efficacy and utility of icatibant in intubated ACEI-induced angioedema patients remain unclear. It may be hypothesized that icatibant administration in an intubated patient may decrease time to extubation and intensive care unit length of stay compared to alternative interventions.

The aim of this article is to share real-life clinical experience of three cases in which patients received icatibant after respiratory intervention, such as intubation and tracheostomy, as a result of ACEI-induced angioedema. In addition, a review of available literature describing other clinical experiences with icatibant utilization in intubated patients is discussed.

## 2. Methods

A literature review using articles published through April 2017 from PubMed and MEDLINE databases was conducted using the search terms* icatibant, angiotensin-converting enzyme induced angioedema*, and* intubation*. Search results were limited to English-language studies conducted on humans. Studies, case reports, and case series describing patients who received icatibant after receiving respiratory interventions were included.

## 3. Case Reports

The three patient cases include one female and two male patients who were intubated due to respiratory distress or failure from ACEI-induced angioedema. Their clinical course leading up to icatibant administration and response thereafter is described (Table 1).

### 3.1. Case  1

A 60-year-old African American female with hypertension presented to the ED with a chief complaint of sudden difficulty in breathing and swallowing after experiencing shortness of breath and coughing for one week. The patient had been on lisinopril therapy for 6 years and 9 months, with a recent increase in dose to 40 mg only two months prior to ED presentation. Home medications also included amlodipine 10 mg daily. The patient was given diphenhydramine en route to the ED and treated with epinephrine nebulization, dexamethasone 10 mg IV, famotidine 20 mg IV, and epinephrine 0.5 mg IM injection upon arrival. Approximately 20 minutes after ED arrival, the patient deteriorated with respiratory failure and required intubation due to severe macroglossia and lip swelling with facial fullness. Her lungs were clear to auscultation. The patient subsequently received two units of fresh frozen plasma (FFP), IV ranitidine, diphenhydramine, and methylprednisolone without improvement. The decision was then made to administer icatibant. Icatibant 30 mg was administered subcutaneously approximately 12 hours after presenting to the ED. Subjective improvement in lip, throat, and tongue edema was not noted until approximately 20 hours after icatibant administration. Steroid therapy was weaned over several days before a laryngoscopy was performed on fourth day of hospitalization. The laryngoscopy showed marked improvement in airway edema and the patient was then successfully extubated. The patient was subsequently downgraded from the intensive care unit on day 5 and discharged from the hospital on seventh day of hospitalization.

### 3.2. Case  2

A 52-year-old African American male with a history of hypertension presented to the ED with complaints of chest pain, difficulty in swallowing and talking, shortness of breath, sensations of “throat closing,” and swelling of the tongue, mouth, and eyes. Per patient report, the swelling had started approximately four hours prior to ED arrival. Upon chart review, the patient was found to have also presented to the ED two days earlier with similar symptoms of lip swelling. During that ED presentation, the patient was treated with a prednisone burst before being sent home with amoxicillin. The patient was noted to have been on lisinopril for hypertension for nearly 3 years, but patient instructions to discontinue lisinopril were not found in the electronic medical record. Upon the subsequent presentation to the ED, the patient was noted to have labored breathing and massive macroglossia without visualization of the uvula on physical exam. He was immediately treated with IM epinephrine, IV diphenhydramine, dexamethasone, and famotidine. Minutes later, the patient became unresponsive and required cardiopulmonary resuscitation. The patient was successfully intubated after multiple attempts due to a difficult airway. Two units of FFP were subsequently administered and the patient continued treatment on IV methylprednisolone, diphenhydramine, famotidine, and albuterol/ipratropium nebulization. Icatibant 30 mg was administered subcutaneously approximately 11 hours after ED presentation and 15 hours after symptom onset. Subjective improvement of lip and eye swelling was noted within 22 hours after icatibant administration. The patient was extubated and downgraded from the intensive care unit on fifth day and discharged on sixth day of hospitalization.

### 3.3. Case  3

A 62-year-old Caucasian male with a history of hypertension presented to the ED with progressive macroglossia and muffled voice which began approximately five hours earlier. Initially, the patient denied shortness of breath but began to develop dyspnea upon arriving to the ED, particularly while supine. Per patient recall, he had experienced a similar episode several years ago and was told that he had an unknown medication allergy. The patient had been on lisinopril for hypertension management for four years. Home medications also included amlodipine 10 mg daily. Upon arrival, the patient was treated with IM epinephrine, IV dexamethasone, diphenhydramine, and famotidine. He was subsequently taken to the operating room, where a laryngoscopy revealed evidence of significant compromised airway with posterior supraglottic edema, trace edema of the epiglottis, prominent base of tongue mucosa, and uvula hydrops. An awake fiberoptic nasal intubation was then performed. After intubation, the patient continued treatment on methylprednisolone 80 mg IV. The patient then received one subcutaneous dose of icatibant 30 mg approximately 11 hours after ED presentation and 16 hours after symptom onset. Subjective improvement of tongue and submandibular soft tissue swelling was noted approximately 11 hours after icatibant administration. A cuff leak test was attempted one day after icatibant administration to no avail. On third day of hospitalization, a cuff leak was present and the patient was successfully extubated. The patient was then downgraded from the intensive care unit on fourth day and discharged on fifth day of hospitalization.

## 4. Discussion and Literature Review

No randomized controlled trials have specifically evaluated the utility of icatibant in patients that required respiratory intervention (intubation or tracheostomy) prior to icatibant administration (Table 2). The randomized controlled trial by Straka et al. included three intubated patients treated with icatibant and one intubated patient treated with placebo; there was no statistical difference in the number of intubated patients between groups (*p* = 0.32) [5]. One of the icatibant-treated intubated patients was excluded from final analysis due to sedation and subsequent inability to participate in a visual analog scale. The authors did not specify whether intubation occurred before or after the assigned intervention. Time to extubation was also not described. Overall results found no difference in the time to symptom resolution compared to placebo (Kaplan-Meier curve, *p* = 0.192) [5].

Several other case series and reports have described the use of icatibant in intubated patients for ACEI-induced angioedema. In a case series by Javaud et al. in 2015, one of 62 patients treated with icatibant for ACEI-induced angioedema required immediate tracheal intubation upon presentation to the ED [7]. The age, gender, and ethnicity of this individual and angioedema severity were not specified. The patient was intubated before icatibant injection and successfully extubated on second day after complete resolution of edema [7]. Another case series by Fok et al. in 2015 described four of 13 patients treated with icatibant after requiring intubation upon presenting to the ED with ACEI-induced angioedema [10]. Three of these patients were determined to have type 2 angioedema (angioedema of the floor of the mouth, palate, or oropharynx), while the remaining patient had type 3 angioedema (angioedema of the hypopharynx or larynx). One of the patients with type 2 angioedema received icatibant one hour prior to intubation, while the two other patients received icatibant at the time of intubation. The patient with type 3 angioedema received the dose of icatibant 72 hours after ED presentation per recommendations by immunology consult. Two of the patients were aged >60 years and three of the patients were female, but the ethnicities of each patient were not specified. The duration of ACEI therapy ranged from one day to 20 years. Three of the four patients were successfully extubated within 24 hours of treatment, but these patients were not specified. Median time to first symptom resolution after treatment for these four patients was three hours (range: two to seven hours) [10]. A case report by Charmillon et al. in 2014 described a 65-year-old woman presenting with severe dyspnea, facial edema, and macroglossia without urticaria or pruritus [8]. Her medication history included quinapril and everolimus. After failure of tracheal intubation, a tracheostomy was performed followed by subcutaneous administration of icatibant 30 mg. Nearly a total regression of angioedema was observed in one hour. On third day of hospitalization, the patient was decannulated and discharged from the intensive care unit [8]. In 2012, Illing et al. also described a 62-year-old male presenting with sudden onset airway compromise, drooling, and macroglossia [9]. His medication history included fosinopril for five years. The patient was treated with hydrocortisone and chlorphenamine and epinephrine with no clinical improvement. Icatibant 30 mg was subsequently administered subcutaneously, but drooling and inability to speak persisted, while upper airway edema progressed. The patient was then intubated and transferred to the intensive care unit. He remained intubated and ventilated for 48 hours. The authors did not appreciate any immediate benefit from icatibant administration, describing “slow resolution of angioedema over 48 hours” before achieving extubation [9].

Overall, the majority of previous case series and case reports suggest a benefit with icatibant administration in intubated patients presenting with ACEI-induced angioedema, but this benefit has not been compared against any patient controls [7–10]. Insufficient details by Straka et al. limit any inferences from patients included in a randomized controlled trial [5]. The majority of reports described noticeable improvement of symptoms when icatibant was administered at the time of intubation or shortly afterwards, with nearly complete regression reported as early as one hour in severe angioedema [8]. Additionally, all reports described extubation within 48 hours or decannulation by 72 hours [7–10].

In the three cases described by this report, however, icatibant was administered after intubation and past the recommended 10 hours from symptoms onset by Bas et al. [4]. Time to icatibant administration ranged from 11 to 12 hours after presenting to the ED and from 15 hours to one week after symptom onset. This delayed administration of icatibant may explain the discrepancies observed between responses in these patients compared to patients previously described in the literature. In this case series, onset of improvement ranged from 11 to 22 hours compared to an onset of improvement and resolution as early as one hour described in previous case reports [8]. Time to extubation ranged from three to five days compared to the one to three days that have been previously reported [10]. Patients of this case series were also hospitalized for at least two to four days longer than patients previously described in the literature [8, 9]. Therefore, delayed administration of icatibant outside the recommended 10 hours from symptom onset presents a large limitation of this case series. Furthermore, the recommended 10-hour administration window was determined in the setting of intubation prevention, limiting its applicability in the postintubation setting [4]. The utility of icatibant in intubated patients for improving symptom resolution, reducing time to extubation, and shortening hospitalization remains unclear, but timely administration of icatibant appears essential for improved patient outcomes. These improved outcomes may also manifest as decreased patient morbidity and increased cost savings, but in the United States, this is limited by the cost of icatibant. As of November 2017, the listed average wholesale price of one dose of icatibant 30 mg/3 mL subcutaneous injection in the United States is approximately $12,400 [11]. Considering data that estimates the 2013 median cost of medical intensive care unit hospitalization at approximately $9,000, evidence-based practice and institution specific cost-benefit analyses seem prudent for delivering optimal patient care [12].

## 5. Conclusion

Icatibant administration in intubated patients may be beneficial in decreasing time to extubation and length of intensive care unit stay. In the three cases described, delayed icatibant administration did not appear to elicit a response in intubated patients, which has been described in previous case reports. For clinicians considering icatibant in the treatment of ACEI-induced angioedema, earlier administration upon arrival to the ED or immediately upon arriving to the intensive care unit is strongly advised. The suggested benefit of icatibant in intubated ACEI-induced angioedema patients should be verified by randomized clinical trials and cost-benefit analyses should be performed at individual institutions.

## Figures and Tables

**Table 1:** Summary of patient cases.

Patients, *n*	Case 1	Case 2	Case 3
Characteristics			
Gender	Female	Male	Male
Age, years	60	52	62
Ethnicity	African American	African American	Caucasian
Duration of ACEI therapy, months	81	36	48
Symptoms	Difficulty breathing and swallowing, shortness of breath, coughing	Chest pain, difficulty swallowing and talking, shortness of breath, sensations of “throat closing,” swelling of tongue, mouth, and eyes	Macroglossia, muffled voice, shortness of breath
Clinical course			
Symptom onset to ED presentation	1 week	4 hours	5 hours
ED presentation to intubation, mins	20	“Minutes later”	Immediately
ED presentation to icatibant, hrs	12	11	11
Icatibant administration	After intubation	After intubation	After intubation
Icatibant to first symptom resolution, hrs	20	22	11
Hospital day of extubation	Day 4	Day 5	Day 3
ED presentation to discharge	Day 7	Day 6	Day 5

**Table 2:** Review of previously published cases of icatibant utilization in intubated patients.

	Straka et al. [5]	Javaud et al. [7]	Charmillon et al. [8]	Illing et al. [9]
Study type	Randomized controlled trial	Prospective observation study	Case report	Case report
Patients, *n*	3	1	1	1
Characteristics				
Gender	--	--	Female	Male
Age, years	--	--	65	62
Ethnicity	--	--	Caucasian	--
Duration of ACEI therapy, months	--	--	--	60
Symptoms	--	--	Tongue/facial swelling/severe dyspnea	Drooling/tongue swelling
Clinical course				
Symptom onset to ED presentation	--	--	--	--
ED presentation to intubation, mins	--	--	--	--
ED presentation to icatibant	--	--	--	--
Icatibant administration	--	After intubation	After intubation	Before intubation
Icatibant to first symptom resolution, hrs	--	--	1	“Slow resolution over 48 hours”
Hospital day/time to extubation from icatibant	--	Day 2	Day 3	48 hours
ED presentation to discharge	--	--	Day 3	“A few days later” after extubation
Conclusion	No difference in number of intubated patients between groups or time to symptom resolution compared to placebo	Icatibant use for ACEI-induced angioedema elicits faster symptom relief than placebo	Icatibant recommended for bradykinin mediated angioedema	Icatibant does not always provide rapid and complete resolution of symptoms caused by angioedema

	Fok et al. [10]			

Study type	Case series			
Patients, *n*	4			
Characteristics				
Gender	Female	Male	Female	Female
Age, years	75	49	40	62
Ethnicity	--	--	--	--
Duration of ACEI therapy	20 years	1 day	2 days	4 years
Symptoms	Lip/tongue swelling	Drooping/facial swelling	Sore throat/drooling	Tongue swelling/hoarse voice
Clinical course				
Symptom onset to ED presentation, hrs	1	24	6	2
ED presentation to intubation, hrs	2	2	1	14
ED presentation to icatibant, hrs	72	2	2	14
Icatibant administration	After intubation	At time of intubation	Before intubation	At time of intubation
Icatibant to first symptom resolution, hrs	7	3	3	2
Hospital day/time to extubation from icatibant	Within 24 hours for 3 out of 4 patients			
ED presentation to discharge	--	--	--	--
Conclusions	Icatibant is effective for adrenaline-unresponsive acute upper airway angioedema involving the larynx or oropharynx			
